# The Assessment of Nursing Staff Knowledge and Barriers Regarding Aseptic Techniques in Khartoum Teaching Hospital, Sudan

**DOI:** 10.7759/cureus.45265

**Published:** 2023-09-14

**Authors:** Ahmed A Jarelnape

**Affiliations:** 1 Department of Nursing, Faculty of Applied Medical Sciences, Al Baha University, Al Baha, SAU

**Keywords:** practices, attitude, knowledge, nursing staff, aseptic techniques

## Abstract

Background

Aseptic techniques are crucial in preventing healthcare-associated infections, which are an integral part of standard precautions, and encompass a range of practices designed to safeguard patients from healthcare-associated infections.

Objective

The objective of this study was to evaluate the level of knowledge and identify the barriers faced by nursing staff in implementing aseptic techniques.

Methodology

This study employed a stratified random sampling technique to ensure the representation of the research sample. A cross-sectional, descriptive research design was used to assess the knowledge and barriers of nursing staff in maintaining aseptic techniques in their medical practice at Khartoum Teaching Hospital, Sudan. The nursing staff members were divided into different units, and a proportionate number of participants were randomly selected from each stratum. A total of 83 nursing staff members were recruited for this study. Data collection was conducted using a structured questionnaire specifically designed for this study. The questionnaire consisted of items that assessed the nursing staff's knowledge and barriers to aseptic technique implementation.

Results

The study findings revealed that the mean knowledge score of nursing staff was 14.12, with a median score of 15. The knowledge score had a standard deviation of 3.22. Approximately two-thirds of the nurses (66.3%) had an average level of knowledge, while 33.7% had a below-average level of knowledge. The chi-square analysis indicated a significant association between educational level, years of experience, and knowledge scores (P value=0.010) at a significance level of 0.05. Additionally, 65% of the participants reported facing multiple challenges in maintaining aseptic techniques, including insufficient training, limited resources, and inadequate support.

Conclusion

In conclusion, the evaluation revealed that a significant proportion of participants felt that their unit lacked adequate training and resources for aseptic techniques. Many had observed colleagues not adhering to aseptic practices, and the participants faced multiple challenges in maintaining aseptic techniques, including insufficient training, limited resources, time constraints, and inadequate support.

## Introduction

Despite the progress in medical advancements and treatments, there has been a global rise in the occurrence of hospital-acquired infections (HAIs) [1]. According to the World Health Organization (WHO), the prevalence of HAIs in hospital settings worldwide ranges from 6% to 19.5% [2]. Recent studies have reported HAI prevalence rates of 3.5% in the United States [3]. However, low-resource countries bear a greater burden of HAIs compared to high-income countries [4,5].

A systematic review conducted by the WHO indicated that the prevalence of HAIs is approximately 7.5% in high-income countries and 15.6% in low- and middle-income countries [6]. HAIs not only result in extended hospital stays and increased mortality rates but also contribute to higher healthcare expenses, placing a financial strain on individuals, communities, and nations [7].

As a result, the prevention and management of HAIs have emerged as significant public health priorities [8]. The main sources of hospital-acquired infections (HAIs) have been identified as the contaminated hands of healthcare workers (HCWs) and medical supplies [9]. Failure to properly wash hands between patient interactions can lead to the transfer of bacteria-causing HAIs from one patient to another [10]. The incidence rates of HAIs vary across different clinical departments, with intensive care units (ICU) having the highest infection rates, followed by newborn and burns units, as reported in a study conducted in Norway [11].

The World Health Organization (WHO) identifies various factors contributing to hospital-acquired infections (HAIs), including insufficient environmental hygiene, improper waste disposal practices, inadequate equipment and staffing, overcrowded living conditions, absence of national guidelines, limited knowledge of and poor adherence to basic infection control measures, and inadequate infrastructure [12].

Aseptic techniques, established by the Centers for Disease Control and Prevention (CDC), encompass specific protocols essential for preventing the transmission of disease-causing agents and thereby decreasing the incidence of HAIs [13]. Adherence to standard precautions, including practices such as hand hygiene, wearing protective gowns, proper sanitization of equipment, utilization of facial protection (masks and goggles), safe disposal of sharp objects, appropriate management of medical waste, and proper coughing techniques, should always be observed [14]. Factors such as heavy workloads, lengthy clinical procedures, and skin conditions have been identified as significant barriers to complying with hand hygiene recommendations [15]. Hence, it is essential to examine the knowledge, attitudes, and practices (KAP) of healthcare workers (HCWs) in order to comprehend the factors contributing to noncompliance and to identify strategies for enhancing infection control measures and preventing HAIs [16]. Consequently, the purpose of this study is to evaluate the level of knowledge and identify the barriers faced by nursing staff in implementing aseptic techniques.

## Materials and methods

Study area

The study was conducted in various nursing units of Khartoum Teaching Hospital in Sudan, with a specific focus on evaluating the understanding of aseptic techniques among nursing staff; this study aims to assess their knowledge and identify the obstacles they face in implementing these techniques.

Study design

The study followed a cross-sectional design; this study focused on the nursing staff as the target population, with an estimated sample size of 83 participants. The sample included nursing staff from all units within the hospital. A stratified random sampling technique was employed to ensure the representation of the research sample from various nursing units.

Data collection techniques

A self-administered survey was utilized to collect data, which was designed based on relevant literature and guidelines, which were developed based on previous studies regarding aseptic techniques [16,17]. The content validity of the questionnaire was ensured through expert review, which assessed its clarity, relevance, comprehensiveness, and overall effectiveness. The questionnaire comprised three sections. The first section collected the demographic characteristics of the study participants. The second section consisted of 15 multiple-choice items assessing the knowledge of nursing staff regarding aseptic techniques. Each item had four options, with only one being the correct answer. Correct responses were assigned a score of 1, while no response or an incorrect response received a score of 0. The third section aimed to identify the barriers encountered by nursing staff in implementing aseptic techniques. Ethical approvals and permissions were obtained, and the researchers visited the selected nursing units to explain the purpose of the study to the participants. Informed consent was obtained, ensuring confidentiality, anonymity, and strict maintenance of participant confidentiality throughout the study.

Pilot study

A pilot study involving 10 nurses from Khartoum Teaching Hospital was conducted to test the questionnaire. Feedback on the pilot study results was obtained from the participants through a separate questionnaire. The participants who took part in the pilot study were not included in the main study analysis.

Sample size and sampling technique

A stratified random sampling was used in this study, and the sample size was calculated according to the following equation: n=N/1+N (d2), where n=sample size, N=population size, and d=degree of accuracy desired (the accepted margin of error was 0.05); n=135/1+135(0.05)2=99. Thus, the sample size for this study was initially intended to be 99 nurses. However, out of the 99 nurses who received the questionnaire, 83 nurses completed the survey, resulting in a response rate of approximately 83.8%.

Statistical analysis

The collected data was analyzed utilizing the statistical software Statistical Package for Social Sciences (SPSS) (IBM SPSS Statistics, Armonk, NY). Descriptive statistics were employed to summarize the demographic characteristics of the nursing staff participants. The knowledge scores were calculated based on the questionnaire responses. Inferential statistics, including the chi-square test, were utilized to investigate relationships between variables and determine if any statistically significant differences existed. The reliability of the instrument was tested using Cronbach's alpha=0.79.

Ethical consideration

This study was approved by the ethics committee of Khartoum Teaching Hospital in Sudan (approval number: KTH 2022. 02). Informed consent was obtained from all participants.

## Results

Regarding age distribution, the majority of participants fell within the 20-30 age range, accounting for 39.8% of the total sample. Participants younger than 20 years constituted 21.7%, while those between 31 and 40 years represented 26.5%. The remaining 12% consisted of participants aged 41 years and above. In terms of marital status, the majority of participants were single, comprising 54.2% of the sample. The married participants accounted for 39.8%, while divorced and widowed individuals represented smaller percentages of 4.8% and 1.2%, respectively. When examining gender distribution, female participants were the majority, accounting for 72% of the sample, while males represented 33.7%. Regarding educational attainment, the highest percentage of participants held a bachelor's degree, with 67.5% falling into this category. Diploma holders constituted 13.2%, while those with a master's degree and PhD accounted for 15.7% and 3.6%, respectively. The majority of participants (62.6%) had 1-2 years of experience, while 15.7% had less than one year of experience. Participants with 3-5 years of experience made up 14.5% of the sample, and those with more than six years of experience comprised 7.2%, as represented in Table 1.

**Table 1:** Distribution of the participants' demographic characteristics (n=83)

Demographic characteristic variables		Frequency	Percentage (%)
Gender	Male	28	33.7%
	Female	55	66.3%
Age in years	<20	18	21.7%
	20-30	33	39.8%
	31-40	22	26.5%
	41 years and more	10	12%
Marital status	Single	45	54.2%
	Married	33	39.8%
	Divorced	4	4.8%
	Widowed	1	1.2%
Educational level	Diploma	11	13.2%
	Bachelor's	56	67.5%
	Master's	13	15.7%
	PhD	3	3.6%
Years of experience	Less than one year	13	15.7%
	1-2 years	52	62.6%
	3-5 years	12	14.5%
	More years six years	6	7.2%

Table 2 displays the distribution of correct answers on nursing staff's knowledge of aseptic techniques. It provides insights into their understanding of infection prevention measures. The results indicate that most participants had good knowledge in various areas. For instance, 72.3% correctly identified the main goal of aseptic techniques, and 66.3% knew the recommended handwashing duration. Of the participants, 81.9% understood establishing a sterile field during surgery, and 83.3% knew the steps for donning and doffing personal protective equipment (PPE); 87.9% recognized the frequency of glove changes, and 79.5% understood medical waste handling. Also, 81.9% knew aseptic principles for invasive procedures, and 83.1% understood sterile instrument usage. Furthermore, 90.4% grasped aseptic wound care principles, and 92.8% described proper hand hygiene with hand sanitizers. However, knowledge was relatively lower in some areas. For example, 62.5% identified proper cleaning and disinfection techniques for medical equipment, and 63.9% recognized risks associated with breaches in aseptic technique.

**Table 2:** Distribution of correct answers regarding the knowledge of nursing staff regarding aseptic techniques (n=83)

	Correct answers	
	Frequency	Percentage (%)
What is the purpose of aseptic techniques in healthcare?	60	72.3%
What is the recommended duration for effective handwashing according to standard hand hygiene guidelines?	55	66.3%
What are the proper techniques for cleaning and disinfecting medical equipment to prevent the spread of infections?	68	81.9%
How would you establish and maintain a sterile field during a surgical procedure?	70	83.3%
Which of the following is an example of an aseptic technique?	73	87.9%
How often should you change gloves during patient care to maintain an aseptic technique?	52	62.5%
Can you identify potential risks associated with breaches in aseptic technique?	53	63.9%
How would you handle and dispose of medical waste to ensure infection prevention?	66	79.5%
What are the principles of aseptic technique when inserting a central line or performing other invasive procedures?	68	81.9%
What are the proper techniques for handling and using sterile instruments during procedures?	69	83.1%
What are the principles of aseptic wound care, and how would you maintain a sterile wound environment?	75	90.4%
Can you describe the steps for proper hand hygiene when using an alcohol-based hand sanitizer?	77	92.8%
Which of the following is an example of a non-sterile item?	67	80.7%
When should sterile gloves be worn?	70	84.3%
What should be done if a sterile item comes into contact with a non-sterile surface?	71	85.5%
Overall knowledge	66	78.8%

Regarding barriers to the successful adoption of aseptic practices as identified by the nursing staff, the results indicate that there are perceived barriers in several areas. For example, 84.3% of the participants acknowledged the presence of barriers to creating and maintaining a sterile field during surgical procedures. Similarly, a majority of participants (65.1%) identified resource limitations that affect the availability or quality of sterile equipment and supplies. In terms of cleaning and disinfecting medical equipment and surfaces, 87.9% of the participants recognized the presence of barriers. Additionally, a small percentage of participants (7.2%) identified communication barriers that hinder the dissemination of aseptic technique guidelines and protocols among nursing staff. Administrative or policy barriers that hinder the implementation of aseptic techniques in healthcare facilities were acknowledged by 65.1% of the participants. Maintaining a clean and hygienic environment in patient care areas was perceived as a barrier by 3.6% of the participants. Challenges in ensuring compliance with aseptic technique principles during invasive procedures were identified by 16.9% of the participants. Moreover, 65.1% of the participants recognized barriers to conducting effective training and education programs on aseptic techniques for nursing staff, as represented in Table 3.

**Table 3:** Barriers to the successful adoption of aseptic practices

	Yes	No
	n (%)	n (%)
Are there barriers to creating and maintaining a sterile field during surgical procedures?	13 (15.7)	70 (84.3)
Are there any resource limitations that affect the availability or quality of sterile equipment and supplies?	54 (65.1)	29 (34.9)
Are there barriers to effectively cleaning and disinfecting medical equipment and surfaces?	10 (12.1)	73 (87.9)
Are there any communication barriers that hinder the dissemination of aseptic technique guidelines and protocols among nursing staff?	6 (7.2)	77 (92.8)
Are there any administrative or policy barriers that hinder the implementation of aseptic techniques in healthcare facilities?	54 (65.1)	29 (34.9)
Are there barriers to maintaining a clean and hygienic environment in patient care areas?	3 (3.6)	80 (96.4)
Are there any challenges in ensuring compliance with aseptic technique principles during invasive procedures?	14 (16.9)	69 (83.1)
Are there barriers to conducting effective training and education programs on aseptic techniques for nursing staff?	54 (65.1)	29 (34.9)

Table 4 shows the relationship between demographic characteristics and the knowledge of nursing staff regarding aseptic techniques. The findings reveal that age is not significantly associated with knowledge scores (chi-square=1.235, P value=0.570). This suggests that age does not play a significant role in determining the level of knowledge regarding aseptic techniques among the nursing staff. In contrast, there is a significant relationship between educational level and knowledge scores (chi-square=1.129, P value=0.010). This indicates that the educational level achieved by the nursing staff has a significant influence on their knowledge of aseptic techniques. Participants with higher educational levels, such as a master's and PhD, tend to exhibit higher knowledge scores compared to those with lower educational levels, such as a diploma or bachelor's degree. Years of experience also demonstrate a significant relationship with knowledge scores (chi-square=1.131, P value=0.010). The results suggest that the number of years of experience as a nurse influences the level of knowledge regarding aseptic techniques. Participants with more years of experience tend to have higher knowledge scores compared to those with fewer years of experience.

**Table 4:** The relationship between demographic characteristics and the knowledge of nursing staff regarding aseptic techniques (n=83)

Demographic characteristic variables	Level of the knowledge score			Chi-square	P value
	Below median	Above median	Poor		
Age in years				1.235	0.57
<20	6 (7.2%)	7 (4.8%)	5 (6%)		
20-30	10 (12%)	13 (15.7%)	10 (12%)		
31-40	6 (7.2%)	8 (9.6%)	8 (9.6%)		
41 years and more	3 (3.6%)	4 (4.8%)	3 (3.6%)		
Educational level				1.129	0.01
Diploma	2 (2.4%)	2 (2.4%)	7 (4.8%)		
Bachelor's	24 (28.9%)	22 (26.5%)	10 (12%)		
Master's	7 (4.8%)	5 (6%)	1 (1.2%)		
PhD	2 (2.4%)	1 (1.2%)	0 (0%)		
Years of experience				1.131	0.01
Less than one year	4 (4.8%)	6 (6.2%)	3 (3.6%)		
1-2 years	18 (21.7%)	23 (27.7%)	11 (13.2%)		
3-5 years	5 (6%)	5 (6%)	2 (2.4%)		
More years six years	2 (2.4%)	3 (3.6%)	1 (1.2%)		

## Discussion

Aseptic techniques encompass a series of protocols and measures implemented in healthcare settings to prevent the transmission and proliferation of infections. These techniques are designed to uphold a sterile and germ-free environment during medical procedures, surgeries, and patient care interventions. The findings of this study shed light on the understanding and challenges faced by nursing staff concerning a crucial aspect for healthcare practitioners: aseptic techniques.

The findings of the study indicate that age does not have a significant relationship with knowledge scores. This means that the age of the nursing staff does not appear to influence their level of knowledge regarding aseptic techniques. This finding aligns with a previous study that has shown no significant correlation between age and knowledge scores related to infection control practices [14]. On the other hand, the study identified significant associations between educational level and years of experience with knowledge scores. Participants with advanced educational qualifications, such as a master's or PhD, exhibited higher knowledge scores in comparison to those with lower educational levels, such as a diploma or bachelor's degree. This finding aligns with previous research indicating a positive correlation between higher education and a stronger understanding of infection control practices [18]. The results showed that there is a significant association between educational level, years of experience, and knowledge scores (P value=0.010) at a significance level of 0.05. This finding is supported by previous research that has shown a positive relationship between years of experience and the knowledge of infection control practices [19].

The results indicate that the participants demonstrated varying levels of knowledge regarding different aspects of aseptic techniques. The participants displayed a reasonably good level of knowledge regarding aseptic techniques, as indicated by the 78.8% overall knowledge score. However, the variation in percentages for individual questions suggests that there may be areas where further education or reinforcement of knowledge is necessary to ensure a comprehensive understanding of aseptic practices among the nurse staff. This result is supported by a study by Dimitriadou et al. (2022) that assessed the knowledge of nursing staff regarding aseptic techniques in an intensive care unit (ICU) setting. The findings revealed knowledge gaps in areas such as hand hygiene, use of personal protective equipment, and prevention of central line-associated bloodstream infections; this study emphasized the need for targeted educational interventions to enhance nurses' knowledge and improve compliance with aseptic practices [20].

The majority of participants in the study provided correct answers regarding the objective of aseptic techniques and their importance in preventing the transmission of infections, accounting for 72.3%. This confirms that the participants have a proper understanding of it. This result is not consistent with a study conducted by Tambe et al., which revealed lower knowledge levels (20%) on the same topic [21].

Regarding the resource limitations and staff training that affect the availability or quality of sterile equipment and supplies, this result was supported by a study whose results suggest that resource limitations affecting the availability or quality of sterile equipment and supplies are identified barriers to aseptic practices [22]. The majority of the study participants (84.3%) indicated that there are obstacles to the optimal application of aseptic techniques; this finding was supported by a study by Gaines et al., who found that there are perceived barriers to creating and maintaining a sterile field during surgical procedures [23]. The distribution of years of experience among the participants provides insights into the level of expertise and professional background of the individuals involved in the study regarding knowledge about aseptic technique. The results of the study showed that participants with varying years of experience can bring different perspectives, knowledge, and skills to the study. Those with more years of experience may have a deeper understanding of the subject matter and potentially contribute valuable insights based on their expertise. This result is supported by a previous study that has shown a positive correlation between years of experience and professional expertise [16].

The study results showed that the majority of participants were females, accounting for 73% of the sample, with ages ranging from 20 to 30 years. This finding was consistent with a study conducted by Jarelnape, where the female gender constituted the majority [24].

The educational background of the participants is an important factor to consider when interpreting the findings of the study. The diverse educational levels among the participants may contribute to variations in their knowledge, expertise, and perspectives on aseptic techniques. This diversity can enrich the discussions and enhance the validity of the study's conclusions. This finding supports a study on the education and training of undergraduate nursing students in sterile technique [25].

There are some limitations to this study such as sample size and representativeness. The study might have included a relatively small sample size from a specific hospital in Sudan. The generalizability of the findings may be limited to other settings or populations due to certain factors. The study likely employed a cross-sectional design, which offers a snapshot of knowledge at a specific moment in time. Additionally, it should be noted that the study was conducted at a single site. Conducting the study in a single hospital might limit the variability in knowledge levels and challenges faced by nursing staff, potentially affecting the generalizability of the findings to other healthcare facilities.

## Conclusions

In conclusion, the study findings revealed that the mean knowledge score of nursing staff regarding aseptic techniques was 14.12, with a median score of 15. The standard deviation of the knowledge scores was 3.22. Approximately two-thirds of the nurses (66.3%) demonstrated an average level of knowledge, while 33.7% had a below-average level of knowledge. The chi-square analysis indicated a significant association between educational level, years of experience, and knowledge scores (P value=0.010) at a significance level of 0.05. Furthermore, 65% of the participants reported encountering various challenges in maintaining aseptic techniques, including insufficient training, limited resources, and inadequate support.

## Appendices

Questionnaire

Questionnaire about the assessment of the knowledge of nursing staff regarding aseptic techniques in Khartoum Teaching Hospital, Sudan.

The aim of this study is to evaluate the level of knowledge and identify the barriers faced by nursing staff in implementing aseptic techniques in Khartoum Teaching Hospital, Sudan.

Your responses will be kept strictly confidential and will be used for research purposes only.

1. Do you agree to take part?

Yes. I agree to participate

No.

Date: ........................................................ Time: .....................................................

Serial number: ....................................... Unit: ......................................................

Section 1

Demographic characteristics of the study participants

2. Gender

Male

Female

3. Age

<20

20-30

31-40

41 years and more

4. Marital status

Single

Married

Divorced

Widowed

5. Educational level

Diploma

Bachelor's

Master's

PhD

6. Experience

Less than one year

1-2 years

3-5 years

More years six years

Section 2

Knowledge among nursing staff regarding aseptic techniques.

Instructions: Please answer the following questions to the best of your knowledge. There is only one correct answer for each question. Circle the appropriate answer.

1. What is the purpose of aseptic techniques in healthcare?

A. To promote cleanliness

B. To prevent the spread of infection

C. To enhance patient comfort

D. To reduce healthcare costs

2. What is the recommended duration for effective handwashing according to standard hand hygiene guidelines?

A. Five seconds

B. 10 seconds

C. 20 seconds

D. 30 seconds

3. What are the proper techniques for cleaning and disinfecting medical equipment to prevent the spread of infections?

A. Wipe with a dry cloth

B. Rinse with water

C. Use a disinfectant according to manufacturer's instructions

D. Use soap and water

4. How would you establish and maintain a sterile field during a surgical procedure?

A. Wipe the area with disinfectant

B. Use sterile gloves only

C. Cover the area with a sterile drape

D. Clean the area with soap and water

5. Which of the following is an example of an aseptic technique?

A. Washing hands with soap and water

B. Using sterile gloves during a surgical procedure

C. Wearing a face mask in the patient's room

D. Cleaning the patient's room with disinfectant

6. How often should you change gloves during patient care to maintain an aseptic technique?

A. Once a day

B. Every two hours

C. Whenever they become visibly soiled

D. Once a week

7. Can you identify potential risks associated with breaches in aseptic technique?

A. No risks involved

B. Increased risk of infection

C. Improved patient outcomes

D. Cost savings

8. How would you handle and dispose of medical waste to ensure infection prevention?

A. Dispose of it in regular trash bins

B. Place it in biohazard bags or containers

C. Flush it down the toilet

D. Leave it in patient rooms for later disposal

9. What are the principles of aseptic technique when inserting a central line or performing other invasive procedures?

A. Minimize traffic in the area and use non-sterile gloves

B. Maintain a clean environment and use sterile gloves and equipment

C. Touch non-sterile surfaces and use regular hand hygiene practices

D. Use clean gloves and avoid using sterile equipment

10. What are the proper techniques for handling and using sterile instruments during procedures?

A. Touch the instruments with bare hands

B. Use a clean cloth to handle instruments

C. Use sterile gloves and avoid touching non-sterile surfaces

D. Use gloves and rinse instruments with water before use

11. What are the principles of aseptic wound care, and how would you maintain a sterile wound environment?

A. Keep the wound open and exposed

B. Clean the wound with tap water

C. Apply sterile dressings and maintain a clean environment

D. Use non-sterile gloves during wound care

12. Can you describe the steps for proper hand hygiene when using an alcohol-based hand sanitizer?

A. Apply a small amount and rub hands together until dry

B. Rinse hands with water and then apply sanitizer

C. Apply sanitizer to dirty hands and then wash with soap and water

D. Do not use sanitizer and only use soap and water for hand hygiene

13. Which of the following is an example of a non-sterile item?

A. Sterile gloves

B. Sterile gauze

C. Sterile syringe

D. Non-sterile dressing

14. When should sterile gloves be worn?

A. During routine patient care

B. When handling contaminated materials

C. Only during surgical procedures

D. Never

15. What should be done if a sterile item comes into contact with a non-sterile surface?

A. Rinse it with water

B. Wipe it with a clean cloth

C. Discard it and obtain a new sterile item

D. Use it as long as it appears clean

Section 3

Table 5 shows the barriers encountered by nursing staff in implementing aseptic techniques.

**Table 5:** Barriers encountered by nursing staff in implementing aseptic techniques Please indicate your level of agreement with the following statements on a scale from 1 to 5, where 1 represents "strongly disagree" and 5 represents "strongly agree" (please put a "P" in the appropriate box).

Perceived barriers	Strongly disagree	Disagree	Neutral	Agree	Strongly agree
The lack of adequate training and education about aseptic techniques					
Insufficient resources (e.g., funding, equipment, and staffing) to support the implementation of aseptic techniques					
The lack of clear guidelines or protocols for implementing aseptic techniques					
Time constraints and competing priorities that hinder the implementation of aseptic techniques					
Limited support from organizational leadership for the implementation of aseptic techniques					
Inadequate communication and coordination among healthcare team members regarding aseptic techniques					
The lack of awareness regarding aseptic techniques					
Environmental factors, such as inadequate ventilation, overcrowded patient rooms, or poor cleanliness					
The lack of effective cleaning and disinfecting of medical equipment and surfaces					
